# Supplementary material for: ClpP/ClpX deficiency impairs mitochondrial functions and mTORC1 signaling during spermatogenesis
Source: Commun Biol. 2023 Oct 5;6:1012. doi: 10.1038/s42003-023-05372-2 (PMC10556007; doi:10.1038/s42003-023-05372-2)
Supplement: Supplementary file 2 — Supplementary Information [file 42003_2023_5372_MOESM2_ESM.pdf]

Figure S1

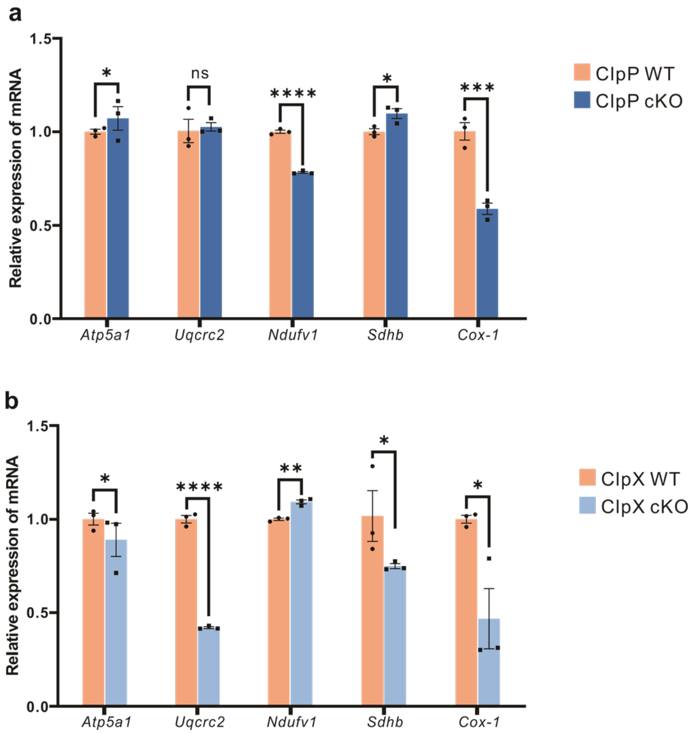

Fig. S1.

The mRNA level of respiratory chain genes in ClpP/ClpX cKO spermatocytes. The spermatocytes from (a) *Clpp*$^{fl/fl}$ mice and *Clpp*$^{fl/fl}$*;S8-Cre* mice or (b) *Clpx*$^{fl/fl}$ mice and *Clpx*$^{fl/fl}$*;S8-Cre* mice were isolated before the RNA were extracted. The mRNA expression levels were measured by RT-qPCR assay. *: p<0.05, **: p<0.01, ***: p<0.001.

Figure S2

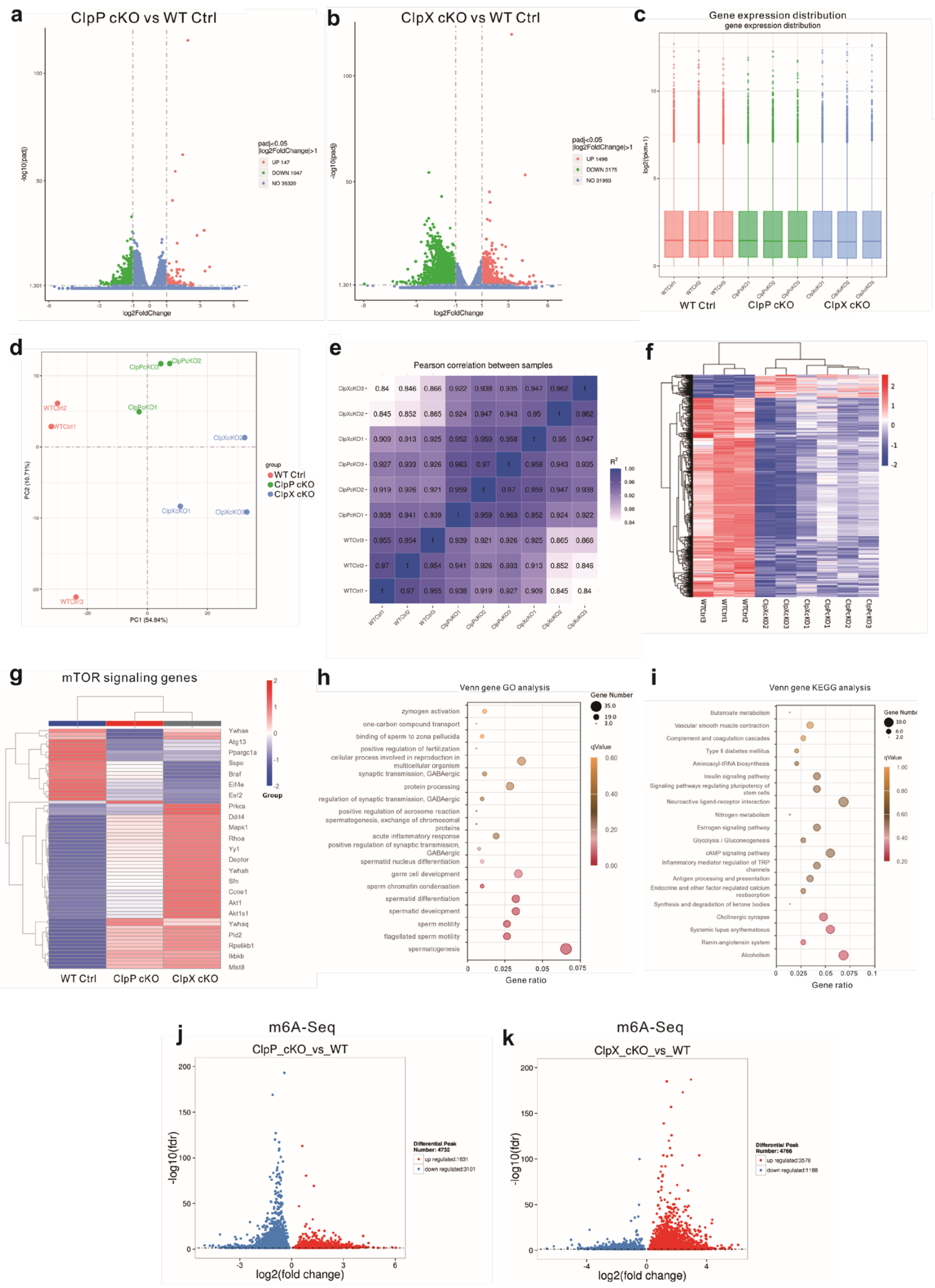

Fig. S2.

The RNA sequencing and m6A sequencing analysis of spermatocytes in WT control, ClpP cKO and ClpX cKO groups. (a-b) Volcano plots of RNA-seq from ClpP cKO and ClpX cKO spermatocytes comparing with controls. Green plots and red plots represent down-regulated genes and up-regulated genes, respectively. (c) gene expression distribution among sequenced samples. (d) Principal component analysis of sequenced samples. (e) Pearson correlation analysis amongsequenced samples. (f) Heatmap and cluster analysis of 1005 common genes obtained from Venn analysis. (g) Heatmap and cluster analysis of mTOR signaling genes expression. (h-i) Dot plots of GO and KEGG analysis for 1005 common genes. (j-k) Volcano plots of m6A-seq data from ClpP cKO and ClpX cKO spermatocytes comparing with controls, blue plots and red plots represent down-regulated genes and up-regulated genes, respectively.

Figure S3

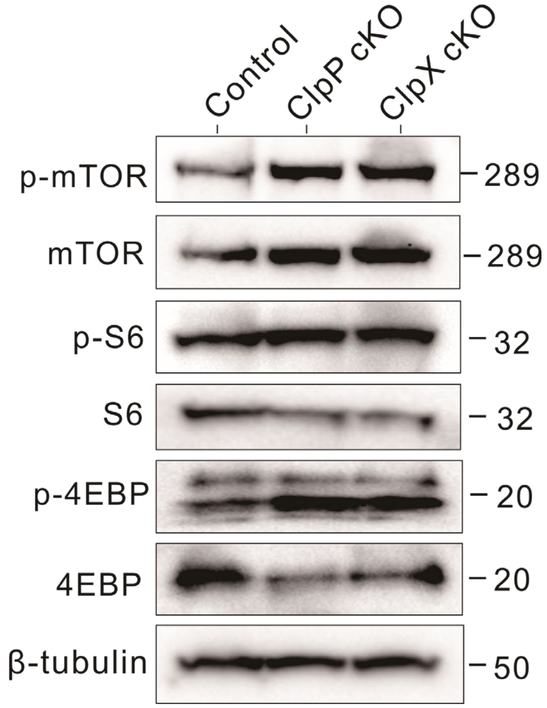

Fig. S3.

The mTORC1 signaling was activated in ClpP/ClpX cKO spermatocytes. The spermatocytes from control mice and ClpP/ClpX cKO mice were isolated before the proteins were extracted. The protein expression level of pan-mTOR, phospho-mTOR, and two substrates of mTORC1 signals, including S6 and 4EBP, as well as their phosphorylated proteins were determined via western blot analysis. β-tubulin acts as a marker for controlling the protein loading for each lane.

Figure S4

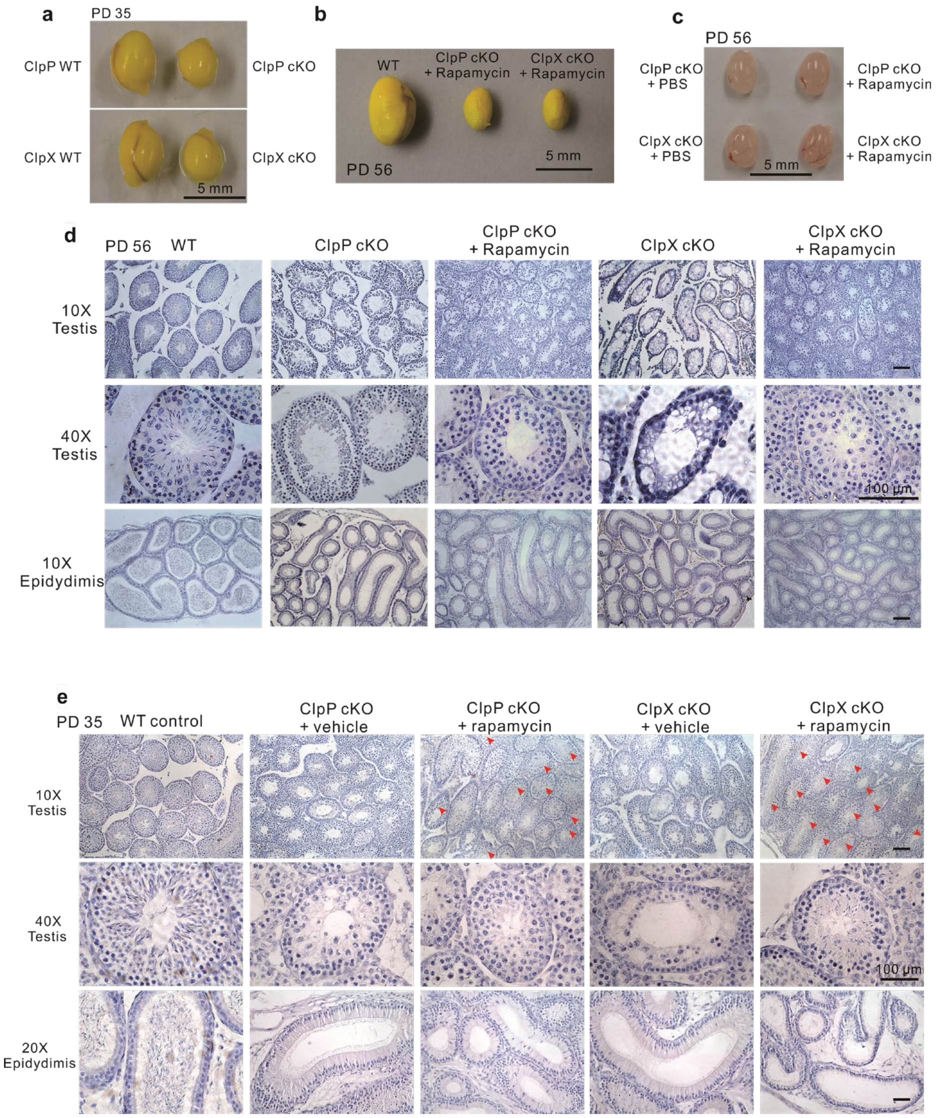

Fig. S4.

Morphology analysis showed rapamycin treatment helps ClpP/ClpX cKO germ cell in meiotic process. (a) The morphology of ClpP/ClpX cKO testes and related controls at PD 35. (b) The morphology of testes from rapamycin injected ClpP/ClpX cKO mice (i.p. injection from PD 14- PD 56, every other day) and WT testis at PD 56. (c) The morphology of testes from PBS or rapamycin injected ClpP/ClpX cKO mice (i.p. injection from PD 14- PD 56, every other day) at PD 56. Scale bars are 5 mm. (d) Histological analysis of testes and epididymis from WT control, ClpP/ClpX cKO and rapamycin injected ClpP/ClpX cKO mice at PD 56. (e) Histological analysis of testes and epididymis from WT control, PBS injected ClpP/ClpX cKO and rapamycin injected ClpP/ClpX cKO mice at PD 35. Red arrow heads indicate elongated spermatozoa in seminiferous tubules of rapamycin treated ClpP/ClpX cKO mice. Scale bars are 100 μm.

Figure S5

The uncropped blots of Fig.1b

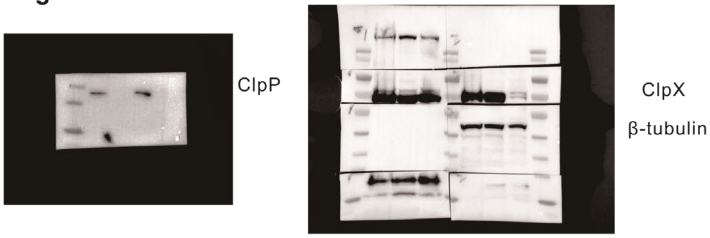

The uncropped blots of Fig.8d

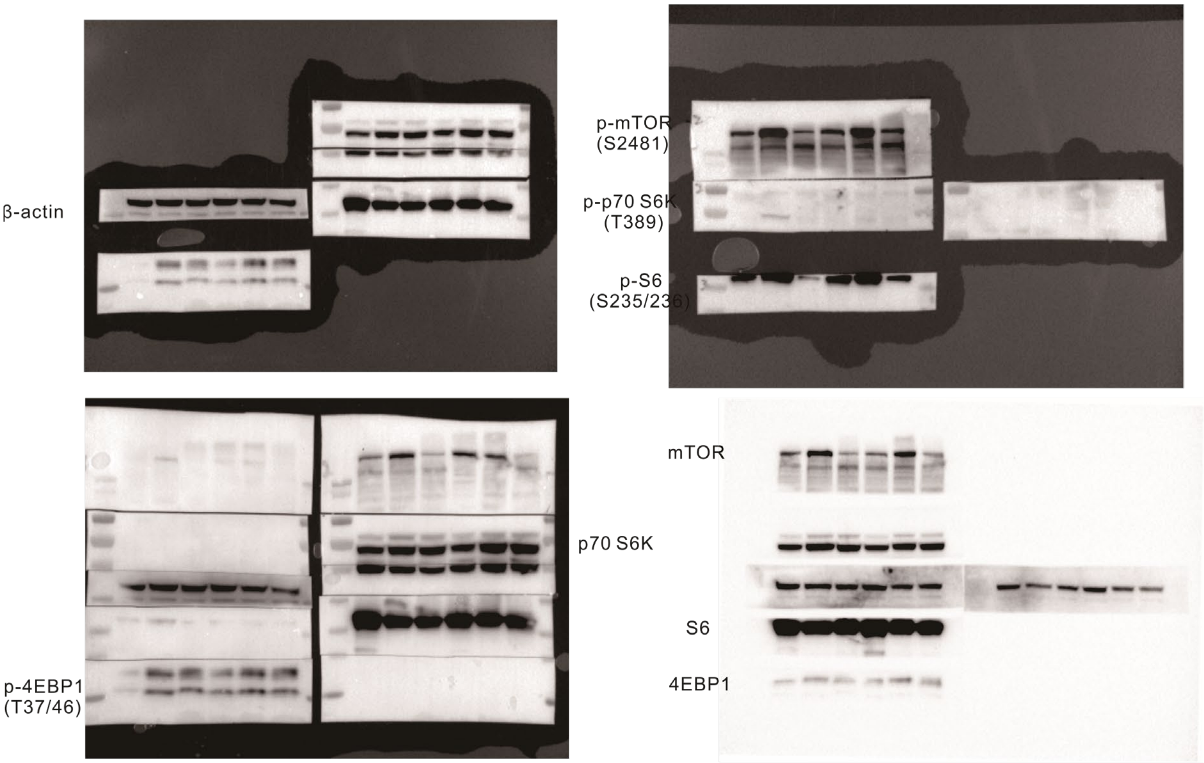

The uncropped blots of Fig.S3

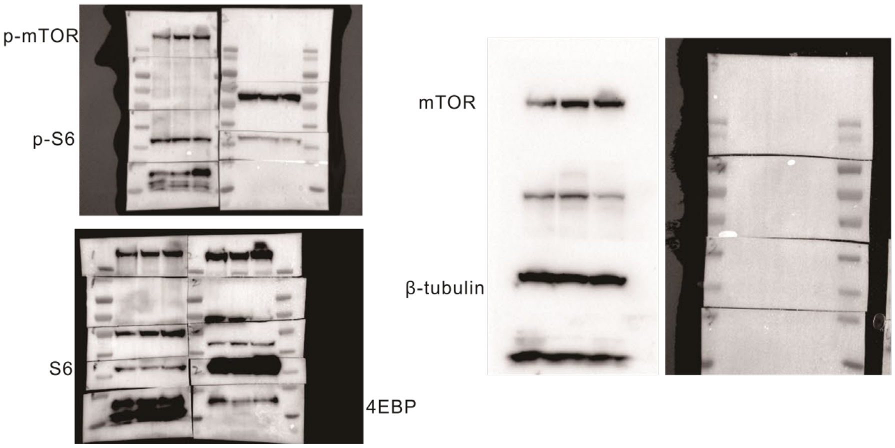

Table S1.
Primary Antibody List

Primary antibodies	Host	Dilution rate	Catalog No.	Manufacturer	Country
Anti-gamma H2A.X (phosphor Ser139)	Rabbit	1:300	ab81299	Abcam	Cambridge, UK
Anti-Sycp3	mouse	1:300	ab181746	Abcam	Cambridge, UK
Anti-cleaved-PARP	Rabbit	1:200	#94885	Cell Signaling Technology	MA, USA
Anti-H1t	Rat	1:100	---------	Custom made by Dai·An	Wuhan, China
Anti-cleaved-caspase 3	Rabbit	1:200	#9664	Cell Signaling Technology	MA, USA
Anti-TRF1	Rat	1:200	ab192629	Abcam	Cambridge, UK
Anti-MLH1	Mouse	1:200	554073	BD	NJ, USA
Anti-alpha Tubulin	Rabbit	1:300	ab52866	Abcam	Cambridge, UK
Anti-beta Tubulin	Rabbit	1:300	#2146S	Cell Signaling Technology	MA, USA
Anti-phospho S6	Rabbit	1:1000	#4856	Cell Signaling Technology	MA, USA
Anti-S6	Rabbit	1:1000	#2217	Cell Signaling Technology	MA, USA
Anti-phospho S6K	Rabbit	1:300	#9234	Cell Signaling Technology	MA, USA
Anti-S6K	Rabbit	1:1000	#2708	Cell Signaling Technology	MA, USA
Anti-phospho 4EBP1	Rabbit	1:1000	#2855	Cell Signaling Technology	MA, USA
Anti-4EBP1	Rabbit	1:1000	#9452	Cell Signaling Technology	MA, USA
Anti-phospho mTOR (Ser2481)	Rabbit	1:1000	#2974	Cell Signaling Technology	MA, USA
Anti-mTOR	Rabbit	1:1000	#2983	Cell Signaling Technology	MA, USA
Anti-β-tubulin	Rabbit	1:2000	#2146	Cell Signaling Technology	MA, USA
Anti-ClpP	Rabbit	1:1000	#14181	Cell Signaling Technology	MA, USA
Anti-ClpX	Rabbit	1:1000	ab168338	Abcam	Cambridge, UK

Table S2.

Secondary Antibody List

Secondary antibodies	Host	Dilution rate	Cat	Manufacturer	Country
Alexa Fluor Plus 488-tagged goat anti-mouse IgG (H+L) highly cross-adsorbed antibody	Goat	1:500	A32723	Invitrogen	MA, USA
Alexa Fluor Plus 488-tagged goat anti-rabbit IgG (H+L) highly cross-adsorbed antibody	Goat	1:500	A32731	Invitrogen	MA, USA
Alexa Fluor Plus 594-tagged goat anti-mouse IgG (H+L) highly cross-adsorbed antibody	Goat	1:500	A32742	Invitrogen	MA, USA
Alexa Fluor Plus 594-tagged goat anti-rabbit IgG (H+L) highly cross-adsorbed antibody	Goat	1:500	A32740	Invitrogen	MA, USA
Alexa Fluor Plus 488-tagged goat anti-rat IgG (H+L) highly cross-adsorbed antibody	Goat	1:500	A48262	Invitrogen	MA, USA
HRP conjugated goat anti-mouse IgG (H+L) cross-adsorbed antibody	Goat	1:5000	G-21040	Invitrogen	MA, USA
HRP conjugated goat anti-rabbit IgG (H+L) cross-adsorbed antibody	Goat	1:5000	G-21234	Invitrogen	MA, USA

Table S3.
The sequence of gRNAs and primers

Name	Sequence (PAM region underlined)
Clpp gRNA1	5'-AAATGTCCTGTCAGCTACATGGG-3'
Clpp gRNA2	5'-CATAGAACCTATGTCGGGCCAGG-3'
Clpx gRNA1	5'-GTTACTTATAGGAATAATCCAGG-3'
Clpx gRNA2	5'-CCGTGGAGCTCAAGGGCAGTTGG-3'
Clpp forward primer	5'- TCTTACCATAGTCTGCTGTTGTCA-3'
Clpp reverse primer	5'- CTACACCTGGTCTGGTTTAGGAAC-3'
Clpx forward primer	5'- GTAAGTCTCCTGATCCAACCT-3'
Clpx reverse primer	5'- TGTCCAGCTAAGGATTCTCATT-3'
Stra8 forward primer	5'-GTGCAAGCTGAACAACAGGA-3'
Stra8 reverse primer	5'- AGGGACACAGCATTGGAGTC-3'
Sdhb forward primer	5'-AATTTGCCATTTACCGATGGGA-3'
Sdhb reverse primer	5'-AGCATCCAACACCATAGGTCC-3'
Uqcrc2 forward primer	5'-AAAGTTGCCCCGAAGGTTAAA-3'
Uqcrc2 reverse primer	5'-AAAGTTGCCCCGAAGGTTAAA-3'
Atp5a1 forward primer	5'-TCTCCATGCCTCTAACACTCG-3'
Atp5a1 reverse primer	5'-CCAGGTCAACAGACGTGTCAG-3'
Ndufv1 forward primer	5'-TTTCTCGGCGGGTTGGTTC-3'
Ndufv 1 reverse primer	5'-GGTTGGTAAAGATCCGGTCTTC-3'
Cox1 forward primer	5'-GTGCTGGGGCAGTGCTGGAG-3'
Cox1 reverse primer	5'-TGGGGCCTGAGTAGCCCGTG-3'
Gapdh forward primer	5'-GGCAAATTCAACGGCACAGT-3'
Gapdh reverse primer	5'-GGCCTCACCCCATTTGATGT-3'
