# Supplementary material for: ClpP/ClpX deficiency impairs mitochondrial functions and mTORC1 signaling during spermatogenesis
Source: Commun Biol. 2023 Oct 5;6:1012. doi: 10.1038/s42003-023-05372-2 (PMC10556007; doi:10.1038/s42003-023-05372-2)
Supplement: Supplementary file 3 — Description of Supplementary Materials [file 42003_2023_5372_MOESM3_ESM.pdf]

Description of Additional Supplementary Files

File name: Supplementary Data 1

Description:All raw data required for statistical analysis.

File name: Supplementary Data 2

Description:A compressed package that summarizes the results of statistical analysis of original data using GraphPad Prism 8 software.
