# Supplementary material for: ClpP/ClpX deficiency impairs mitochondrial functions and mTORC1 signaling during spermatogenesis
Source: Commun Biol. 2023 Oct 5;6:1012. doi: 10.1038/s42003-023-05372-2 (PMC10556007; doi:10.1038/s42003-023-05372-2)
Supplement: Supplementary file 6 — Reporting Summary [file 42003_2023_5372_MOESM6_ESM.pdf]

Corresponding author(s): DBPR COMMSBIO-23-0922C

Last updated by author(s): Sep 13, 2023

Reporting Summary

Nature Portfolio wishes to improve the reproducibility of the work that we publish. This form provides structure for consistency and transparency in reporting. For further information on Nature Portfolio policies, see our Editorial Policies and the Editorial Policy Checklist.

Statistics

For all statistical analyses, confirm that the following items are present in the figure legend, table legend, main text, or Methods section.

n/a	Confirmed	
☐	☒	The exact sample size (*n*) for each experimental group/condition, given as a discrete number and unit of measurement
☐	☒	A statement on whether measurements were taken from distinct samples or whether the same sample was measured repeatedly
☐	☒	The statistical test(s) used AND whether they are one- or two-sided<br>*Only common tests should be described solely by name; describe more complex techniques in the Methods section.*
☐	☒	A description of all covariates tested
☐	☒	A description of any assumptions or corrections, such as tests of normality and adjustment for multiple comparisons
☐	☒	A full description of the statistical parameters including central tendency (e.g. means) or other basic estimates (e.g. regression coefficient) AND variation (e.g. standard deviation) or associated estimates of uncertainty (e.g. confidence intervals)
☐	☒	For null hypothesis testing, the test statistic (e.g. *F*, *t*, *r*) with confidence intervals, effect sizes, degrees of freedom and *P* value noted<br>*Give P values as exact values whenever suitable.*
☒	☐	For Bayesian analysis, information on the choice of priors and Markov chain Monte Carlo settings
☒	☐	For hierarchical and complex designs, identification of the appropriate level for tests and full reporting of outcomes
☒	☐	Estimates of effect sizes (e.g. Cohen's *d*, Pearson's *r*), indicating how they were calculated

Our web collection on statistics for biologists contains articles on many of the points above.

Software and code

Policy information about availability of computer code

Data collection	GraphPad Prism 8 was used for data collection and analysis.
Data analysis	GraphPad Prism 8 was used for data collection and analysis.

For manuscripts utilizing custom algorithms or software that are central to the research but not yet described in published literature, software must be made available to editors and reviewers. We strongly encourage code deposition in a community repository (e.g. GitHub). See the Nature Portfolio guidelines for submitting code & software for further information.

Data

Policy information about availability of data

All manuscripts must include a data availability statement. This statement should provide the following information, where applicable:

- Accession codes, unique identifiers, or web links for publicly available datasets
- A description of any restrictions on data availability
- For clinical datasets or third party data, please ensure that the statement adheres to our policy

All data generated or analyzed during this study are available in the main text or the supplementary materials. The datasets presented in this study can be found in online repositories. The names of the repository/repositories and accession number(s) can be found below: https://www.ncbi.nlm.nih.gov/, GSE220600, GSE221888.

Research involving human participants, their data, or biological material

Policy information about studies with human participants or human data. See also policy information about sex, gender (identity/presentation), and sexual orientation and race, ethnicity and racism.

Reporting on sex and gender	No human research participants in this manuscript.
Reporting on race, ethnicity, or other socially relevant groupings	No human research participants in this manuscript.
Population characteristics	No human research participants in this manuscript.
Recruitment	No human research participants in this manuscript.
Ethics oversight	No human research participants in this manuscript.

Note that full information on the approval of the study protocol must also be provided in the manuscript.

Field-specific reporting

Please select the one below that is the best fit for your research. If you are not sure, read the appropriate sections before making your selection.

☒ Life sciences ☐ Behavioural & social sciences ☐ Ecological, evolutionary & environmental sciences

For a reference copy of the document with all sections, see nature.com/documents/nr-reporting-summary-flat.pdf

Life sciences study design

All studies must disclose on these points even when the disclosure is negative.

Sample size	6 mice each group were sacrificed for collecting data to compare the size of testis and the weight of testes and whole bodies, these testis were further analyzed for tissue histological studies to access the inner structure of testis and epididymis. Three repeats for each group were performed for Western blot assay and qPCR assay. The germ cells which were perfomed meiotic spreads were repeated from three individual mice for each group.
Data exclusions	No data exclusion.
Replication	All attempts at replication were successful.
Randomization	The samples were allocated randomly.
Blinding	We took two investigators were blinded when allocating samples and collecting data.

Reporting for specific materials, systems and methods

We require information from authors about some types of materials, experimental systems and methods used in many studies. Here, indicate whether each material, system or method listed is relevant to your study. If you are not sure if a list item applies to your research, read the appropriate section before selecting a response.

Materials & experimental systems

n/a	Involved in the study	
☐	☒	Antibodies
☒	☐	Eukaryotic cell lines
☒	☐	Palaeontology and archaeology
☐	☒	Animals and other organisms
☒	☐	Clinical data
☒	☐	Dual use research of concern
☒	☐	Plants

Methods

n/a	Involved in the study	
☒	☐	ChIP-seq
☒	☐	Flow cytometry
☒	☐	MRI-based neuroimaging

Antibodies

Antibodies used	Please see supplementary tables for detailed information of antibodies used in this study.

Validation	Please see supplementary tables for detailed information of antibodies used in this study.

Animals and other research organisms

Policy information about studies involving animals; ARRIVE guidelines recommended for reporting animal research, and Sex and Gender in Research

Laboratory animals	All the mice used were of the C57BL/6J background. The ClpP/ClpX conditional knockout mice were generated from C57BL/6J background mice. The detailed information please see Material and Methods part in the manuscript.
Wild animals	Clpp/Clpx floxed C57BL/6J background mice were used as wild animal controls.
Reporting on sex	Male mice were used in all experiments as we focused on male germ cell development.
Field-collected samples	Our study did not include samples colleted from field.
Ethics oversight	All experimental protocols were approved by the ethics committee of the University of Hong Kong-Shenzhen Hospital.(Permit Number: [2021]035 / [2023]035-002).

Note that full information on the approval of the study protocol must also be provided in the manuscript.
